# The *MACROD2* rs6110695 A>G Polymorphism and the Metabolites Indoleacrylic Acid and Butyrylcarnitine Potentially Have Clinical Relevance to WBC Count Prediction

**DOI:** 10.3390/jpm14080889

**Published:** 2024-08-22

**Authors:** Youngmin Han, Minjoo Kim, Hye Jin Yoo

**Affiliations:** 1 Institute for Health Promotion, Graduate School of Public Health, Yonsei University, Seoul 03722, Republic of Korea; ymhan@yonsei.ac.kr; 2 Department of Food and Nutrition, College of Life Science and Nano Technology, Hannam University, Daejeon 34054, Republic of Korea; minjookim@hnu.kr; 3 Institute for Specialized Teaching and Research (INSTAR), Inha University, Incheon 22332, Republic of Korea; 4 Department of Biomedical Science, BK21 FOUR Program in Biomedical Science and Engineering, Inha University, Incheon 22332, Republic of Korea

**Keywords:** WBC count, *MACROD2*, rs6110695 SNP, Korean chip, indoleacrylic acid, butyrylcarnitine

## Abstract

Our previous study suggested that the *Mono-ADP ribosylhydrolase 2* (*MACROD2*) rs6110695 A>G polymorphism is significantly associated with white blood cell (WBC) count in the Korean population. The present study aimed to evaluate the clinical relevance of the *MACROD2* rs6110695 A>G polymorphism for predicting WBC count by utilizing plasma metabolites and a single-nucleotide polymorphism (SNP). Two groups were characterized by *MACROD2* rs6110695 A>G SNP genotypes among 139 healthy subjects based on the genetic information provided in our previous work: rs6110695 AA genotype group (*n* = 129) and rs6110695 AG genotype group (*n* = 10). Plasma global metabolic profiling was performed using ultra-high-performance liquid chromatography–tandem mass spectrometry (UHPLC–MS/MS). To estimate the predictive abilities of WBC count models using the rs6110695 genotype and/or significant differential metabolites, multiple linear regression analysis and receiver operating characteristic (ROC) curve analysis were conducted. The AG genotype had greater WBC-to-apolipoprotein (apo) A-I ratios; counts of WBCs, lymphocytes, monocytes, and granulocytes; monocyte-to-lymphocyte ratio (MLR); and monocyte-to-platelet ratio (MPR) than the AA genotype. In terms of metabolic profile, indoleacetic acid, and butyrylcarnitine levels were considerably distinct between the two groups, and these metabolites were considered to be meaningful prognostic variables for the rs6110695 genotype. Finally, ROC curve analysis demonstrated that the model containing the rs6110695 genotype and the two main metabolites was reliable. The present study revealed that individuals carrying the rs6110695 AG genotype with high plasma indoleacrylic acid and butyrylcarnitine levels might have elevated WBC counts. The rs6110695 genotype and the concentrations of indoleacrylic acid and butyrylcarnitine could contribute to reducing the risk of chronic diseases in the future.

## 1. Introduction

White blood cell (WBC) count is a well-known systemic inflammation marker [1]. Studies have reported that WBC count is associated with chronic diseases such as heart failure, coronary artery disease, hypertension, and type 2 diabetes and can be used as a predictor of chronic diseases [1,2,3,4]. In Korea, chronic disease is a leading cause of death (81% of all-cause death), and related medical expenses are increasing (80% of total medical expenses) [5]. Thus, the prediction of WBC count can help indicate the future risk of chronic disease development.

Research has demonstrated the utility of body fat composition in predicting changes in WBC levels [6] and the potential roles of γ-glutamyltransferase, high-sensitivity C-reactive protein (hs-CRP), interferon (IFN)-γ, dihomo-γ-linolenic acid, and ω-3 polyunsaturated fatty acids as predictors of WBC level change [7]. Moreover, our previous study identified the rs6110695 A>G single nucleotide polymorphism (SNP) as a novel marker related to WBC count in Korean individuals; *Mono-ADP ribosylhydrolase 2* (*MACROD2*), in which this SNP is located, is the candidate gene [8]. The study suggested that an individual’s rs6110695 SNP genotype could predict WBC levels. Unfortunately, basic understanding as well as the clinical relevance of the *MACROD2* rs6110695 A>G polymorphism remain limited.

Metabolites, which are small molecules (<1500 Da) in blood, cells, or tissues, closely reflect phenotypic features as intermediates or end products of metabolism [9]. Internal (e.g., genetic variants) and/or external (e.g., diet, environment, physical activity) factors affect metabolites [9,10,11]; therefore, a set of information on genes and metabolites for a given phenotype can provide understanding of the possible biological processes. In this respect, the present study was designed to obtain plasma metabolite information using ultra-high-performance liquid chromatography–tandem mass spectrometry (UHPLC–MS/MS) to identify rs6110695 SNP-related metabolites that complement its predictive ability for WBC count.

Here, we aimed to validate the clinical relevance of the *MACROD2* rs6110695 A>G polymorphism to WBC count by utilizing metabolites together with the SNP genotype. We expected that a combination of the rs6110695 SNP genotype and metabolic traits would have a better predictive ability for WBC count than using genotype alone.

## 2. Materials and Methods

### 2.1. Study Population

One hundred thirty-nine healthy Korean individuals, recruited at Yonsei University (February 2015–June 2020), were selected for this cross-sectional study. The inclusion criteria were Korean male and female adults (aged between 20 and 80 years) without critical diseases who agreed to participate in this study and provide blood specimens. The exclusion criteria were a history and/or current diagnosis and symptoms of leukemia or immune-related diseases, including autoimmune diseases, cancers, myocardial infarction, stroke, liver diseases, renal disease, pulmonary disease, neurological disease, and mental illness; pregnancy or lactation; and individuals who were unsuitable for participation in this study according to the judgment of the researchers. The purpose of this study was carefully explained to all participants, and they all provided written informed consent. The purpose of this study was thoroughly explained to all study participants, who subsequently provided written informed consent. The study protocol was approved by the Institutional Review Board of Yonsei University and adhered to the Declaration of Helsinki (IRB No.: 7001988-202107-BR-1177-02, Approval date: 20 April 2021).

### 2.2. Blood Sample Collection

Twelve-hour overnight fasting venous blood was collected from the study participants. Serum- and EDTA-treated tubes (BD Vacutainer; Becton, Dickinson and Company, Franklin Lakes, NJ, USA) were used to collect the venous blood samples. After centrifugation (1200 rpm, 20 min, 4 °C), serum and plasma samples were isolated from the blood and stored at −80 °C until analysis.

### 2.3. Anthropometric and Clinical/Biochemical Parameters

Detailed information on the measurements is described in the Supplementary Data (Supplementary Information). Briefly, height (m^2^), weight (kg), waist circumference (cm), hip circumference (cm), and systolic and diastolic blood pressures (BPs) were measured. Body mass index (BMI; kg/m^2^) and the waist-to-hip ratio were calculated.

Glucose and insulin levels were measured using the hexokinase and electrochemiluminescence immunoassay (ECLIA) methods, respectively. Based on the values for glucose and insulin, insulin resistance was calculated using the following equation: Homeostatic model assessment for insulin resistance (HOMA-IR) = insulin × glucose ÷ 405. Adiponectin, which is related to insulin sensitivity, was quantified with a commercial kit.

Regarding lipid profiles and inflammatory markers, triglyceride, total cholesterol, high-density lipoprotein (HDL) cholesterol, interleukin (IL)-1β, IL-2, IL-6, IL-12, IFN-γ, tumor necrosis factor (TNF)-α, and hs-CRP were measured with commercial kits following the manufacturer’s protocol. The Friedewald formula was used to calculate the value of low-density lipoprotein (LDL) cholesterol. Apolipoprotein (apo) A-I and apo B levels were measured via the turbidimetric immunoassay (TIA) method.

Additionally, a HORIBA ABX diagnostic analyzer (HORIBA ABX SAS, Parc Euromedicine, Montpellier, France) was used to analyze the counts of total blood cells, WBCs (lymphocytes, monocytes, and granulocytes) and platelets. The monocyte-to-lymphocyte ratio (MLR), granulocyte-to-lymphocyte ratio (GLR), platelet-to-lymphocyte ratio (PLR), monocyte-to-platelet ratio (MPR), and WBC-to-apo A-I ratio were calculated.

### 2.4. MACROD2 rs6110695 A>G Polymorphism

In our previous study, the rs6110695 SNP, located in the *MACROD2* gene (intron variant), was revealed to have a significant association with WBC count [8]. Details about the genotyping array used to detect this SNP are well described in the Supplementary Data (Supplementary Information). Briefly, the Korean Chip (K-CHIP), developed by the Center for Genome Science, Korea National Institute of Health, Republic of Korea (4845-301, 3000-3031), was used to generate genotype data. Then, quality control of the genotype data and association tests between the SNPs and WBC counts were performed using PLINK ver. 1.07 (http://zzz.bwh.harvard.edu/plink/ (accessed on 25 June 2021)).

### 2.5. Metabolic Profiling

#### 2.5.1. UHPLC–MS/MS Analysis

For the analysis, 100 μL of the plasma was precipitated with 300 μL of cold acetonitrile (Wako Pure Chemical Industries, Osaka, Japan) and centrifuged (13,000 rpm, 15 min, 4 °C). The metabolite-containing supernatant was transferred to new tubes for solvent evaporation under nitrogen gas (N_2_). Then, 100 μL of 10% methanol (J.T. Baker^®^ Chemicals; Avantor Performance Materials, Inc., Radnor, PA, USA) was used to dissolve the dried residue, and the resulting reconstituted samples were filtered with a 0.45 μm polyvinylidene difluoride syringe filter. L-Leucine-1-^13^C (Sigma–Aldrich, Saint Louis, MO, USA) was employed as the internal standard (ISTD). The quality control (QC) sample was prepared by combining all plasma samples and following the same procedure.

Prepared plasma samples were separated on an Acquity UPLC-BEH-C18 column (Waters, Milford, MA, USA) on a Thermo UHPLC system (Ultimate 3000 BioRS; Dionex, Thermo Fisher Scientific, Bremen, Germany) with a 5 μL injection volume and a gradient of two mobile phases in positive electrospray ionization mode (ESI+). The Q Exactive Plus Orbitrap (Thermo Fisher Scientific, Waltham, MA, USA) was coupled with the UHPLC system for molecular detection. Detailed information is provided in the Supplementary Data (Supplementary Information). The QC sample was injected as every 10th sample.

#### 2.5.2. Identification of Metabolites

Raw data from two batches were processed using Compound Discoverer 2.1 software (Thermo Fisher Scientific, San Jose, CA, USA) after assessing the relative peak intensity and retention time (RT) of the ISTD in the QC samples. The spectra were aligned under filter settings, and the intensity deviations were normalized based on the QCs in the program. To integrate the metabolic profiling results into one set, data were normalized using an ISTD. Features detected at less than 80% in all QC samples were considered undesirable and discarded [10]. The remaining features were identified using the online databases [ChemSpider (http://www.chemspider.com (accessed on 16 December 2021)) and mzCloud (https://www.mzcloud.org (accessed on 16 December 2021))] that were employed by the Compound Discoverer software.

### 2.6. Statistical Analysis

SPSS ver. 26.0 (IBM, Armonk, NY, USA) was used for most statistical analyses in the present study. For a comparison of continuous variables between the rs6110695 AA and AG genotypes, the independent *t*-test was performed on normally distributed data or data that followed a normal distribution after logarithmic transformation. In contrast, the Mann–Whitney *U* test was performed for data that did not follow a normal distribution after logarithmic transformation. For descriptive purposes, continuous variables are presented as the mean ± standard error (SE). Fisher’s exact test was used to analyze the sex distributions of the rs6110695 AA and AG genotypes, and the data are presented as numbers and percentages. To identify major metabolites, a volcano plot was generated on MetaboAnalyst 5.0 (https://www.metaboanalyst.ca (accessed on 22 March 2022)). Multiple linear regression analysis was conducted to verify whether each major metabolite was a significant predictor for the rs6110695 genotype. Finally, to estimate the predictive ability of each model (Model 1: rs6110695 genotype (SNP); Model 2: indoleacrylic acid + butyrylcarnitine (major metabolites); Model 3: SNP + major metabolites) for WBC count, receiver operating characteristic (ROC) curve analysis was performed, and a WBC count cutoff value of 5.450, which was derived from our previous study [8], was used for the analysis. All *p* values < 0.05 (two-tailed) were considered to be indicative of significance.

## 3. Results

The present study included 139 study participants in total. Among the participants, 129 were AA genotype individuals, and the remaining were AG genotype individuals. None of the participants had minor homozygotes (GG genotype) for the rs6110695 SNP. A minor allele frequency (MAF) in this study population was 0.0360, comparable with the MAF, which is reported as G = 0.0317 and G = 0.0332 from the Korean Genome Project study and the Korean Reference Genome Database (KRGDB) study, respectively [12]. On a global scale, the MAF of rs6110695 has been documented as 0.1020 according to the 1000 Genomes Project study; in detail, Africans, Europeans, South Asians, and Americans exhibit MAFs of 0.1581, 0.0895, 0.123, and 0.092, respectively [12]. Notably, East Asians in the 1000 Genomes Project study display an MAF of 0.0278 [12]. Consequently, the rs6110695 SNP in this population, including Koreans, appears to manifest at a relatively lower rate than in other populations worldwide.

### 3.1. Anthropometric and Clinical/Biochemical Characteristics

As described above, 129 participants with the AA genotype and the remaining with the AG genotype were divided into AA genotype and AG genotype groups, respectively. As shown in Table 1, no significant differences in anthropometric measurements, lipid profiles, or glucose and insulin resistance-related markers were observed between the AA and AG genotype groups except for the WBC-to-apo A-I ratio; the AG genotype group exhibited a higher WBC-to-apo A-I ratio than the AA genotype group. Given the effects of sex, the variables were separated for male and female subjects. In the male subset, due to the very small sample size, all variables were tested nonparametrically. As a result, none of the variables showed significant differences (Table S1). However, the value of the WBC-to-apo A-I ratio was higher in the AG genotype group than in the AA genotype group (0.06 ± 0.01 and 0.04 ± 0.00, respectively; *p* = 0.235) (Table S1). In the female subset, the results were almost the same as those for the total set (Table S1).

### 3.2. Total Blood Cell Count and Inflammatory Markers

The counts of WBC, lymphocyte, monocyte, and granulocyte, as well as MLR and MPR, were significantly higher in the AG genotype group compared to the AA genotype group (Table 2). On the other hand, the percentage of lymphocytes (% lymphocyte) was lower in the AG genotype group than in the AA genotype group (Table 2). None of the inflammatory markers showed a significant difference between the two groups (Table 2). In the male subset, no differences were observed (Table S2). In contrast, the female subset showed significant differences, as presented in Table S2. Only the significance of MLR disappeared, while other variables, including WBC count, lymphocyte count, monocyte count, granulocyte count, and MPR, remained significant (Table S2).

### 3.3. Differential Metabolites between the Two rs6110695 Genotype Groups

**Figure 1:**
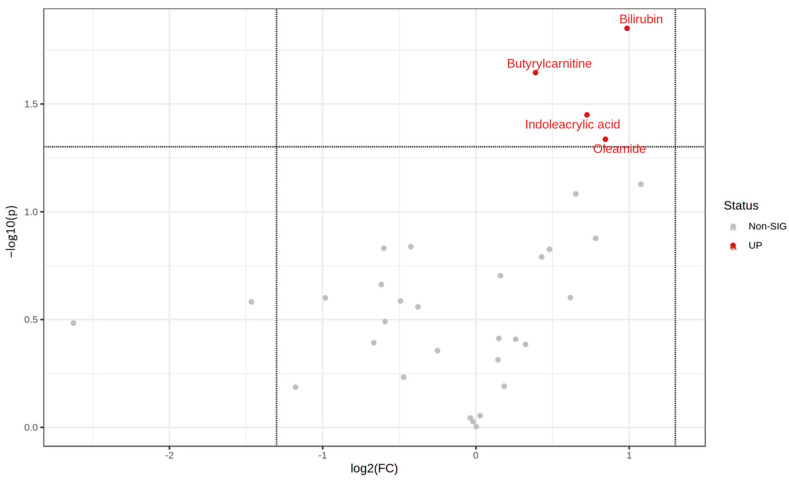
Volcano plot of metabolite profiling data. The x-axis represents each metabolite’s fold change (log_2_ scale) comparing the two groups. The y-axis displays the *p*-value of the ratio fold change for each metabolite.

As described in the methods section, the two batches of metabolic profiling results were combined into one set in the present study. Thirty-three identified metabolites were detected as duplicates (Table S3). A volcano plot was generated using the relative peak intensity of the metabolites to identify the significant differential metabolites between the AG and AA genotype groups (Figure 1). The results showed that bilirubin (log_2_fold change (FC) = 0.986, log_10_*p* = 1.851), butyrylcarnitine (log_2_FC = 0.389, log_10_*p* = 1.645), indoleacrylic acid (log_2_FC = 0.725, log_10_*p* = 1.450), and oleamide (log_2_FC = 0.845, log_10_*p* = 1.337) were significant. 

**Figure 2:**
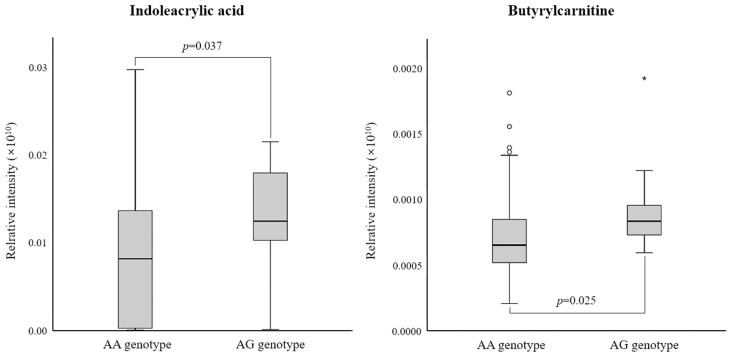
Two major metabolites were significantly different between the rs6110695 genotypes. Mean ± standard error (SE). The *p*-value of each metabolite was derived from independent *t*-tests following logarithmic transformation. *p* < 0.05 was considered to indicate significance.

Among them, indoleacrylic acid (*p* = 0.037) and butyrylcarnitine (*p* = 0.025) were significantly elevated in the AG genotype group compared to the AA genotype group (Figure 2). These two metabolites were revealed to be significant independent predictors of the rs6110695 genotype (indoleacrylic acid: *p* = 0.018, standardized β = 0.200 (95% confidence interval: 0.004–0.041); butyrylcarnitine: *p* = 0.009, standardized β = 0.221 (95% confidence interval: 0.037–0.259)) in multiple linear regression analyses. According to sex, in the male subset, no significant differences were observed for the two metabolites, which might be attributed to the insufficient sample size (too small) (Table S4). In the female subset, indoleacrylic acid and butyrylcarnitine were significantly elevated in the AG genotype group compared to the AA genotype group (*p* = 0.024 and *p* = 0.025, respectively) (Table S4). Finally, consistent with the results for the total set, these metabolites were also identified as significant independent predictors of the rs6110695 genotype in the female subset as well (indoleacrylic acid: *p* = 0.030, standardized β = 0.196 (95% confidence interval: 0.002–0.039); butyrylcarnitine: *p* = 0.011, standardized β = 0.230 (95% confidence interval: 0.034–0.256)).

### 3.4. Evaluation of Prediction Models of WBC Count via ROC Curve Analysis

**Figure 3:**
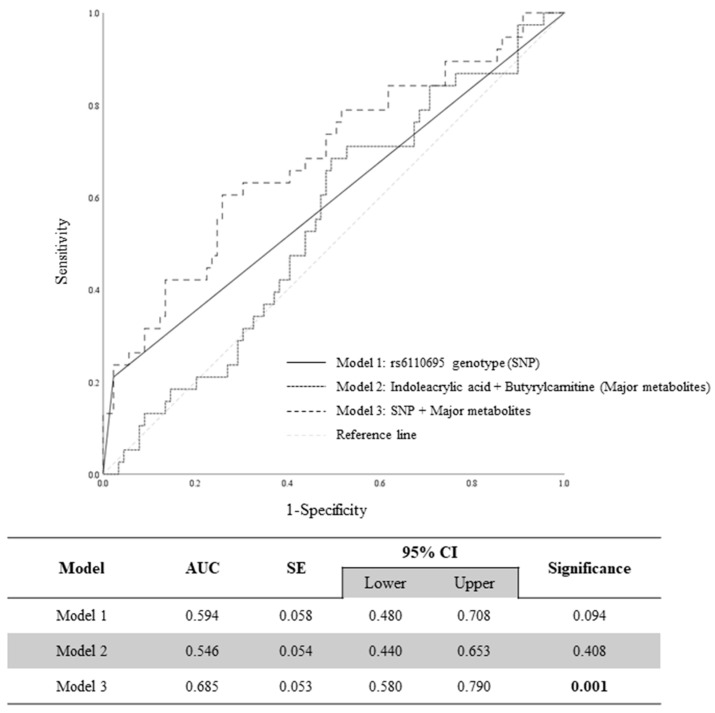
Prediction models for WBC count using the rs6110695 genotype and major metabolites. AUC: area under the curve. CI: confidence interval. SE: standard error.

In our previous study, the threshold of WBC count that best discriminated between the rs6110695 genotypes was 5.450 [8]. Using this cutoff value, the predictive abilities of the three models for WBC count were analyzed by ROC curve. Figure 3 indicates neither Model 1, which consisted of the rs6110695 genotype (SNP), nor Model 2, which included indoleacrylic acid and butyrylcarnitine (major metabolites), were able to substantially predict WBC count. In contrast, Model 3, which included both the SNP and major metabolites, showed a significant predictive ability (68.5%) for WBC count (Figure 3). Similarly, the same results were observed in the female subset (Figure S1). Only Model 3 significantly predicted WBC count; however, it did not exceed 70%, the commonly accepted threshold, indicating somewhat limited predictive ability. Nevertheless, as a foundational approach for predicting WBC count, it still holds potential.

## 4. Discussion

In the present study, two groups were characterized by *MACROD2* rs6110695 A>G SNP genotypes, which are associated with the WBC increase identified in our previous study [8]. Metabolomics newly revealed indoleacrylic acid and butyrylcarnitine to be differential metabolites between the two groups (AA and AG genotype groups). Finally, ROC curve analysis showed that combining the SNP and these two major metabolites could significantly predict WBC levels. Regarding the impact of sex, it should be carefully considered in this study, as it could not be fully elucidated due to the sample size issue.

MACROD2 is a kind of mono ADP-ribosyltransferase with ADP-ribosyl hydrolase activity [13,14]. Therefore, abnormalities in the *MACROD2* gene alter DNA stability, DNA repair, and sensitivity to DNA damage [15]. Chang et al. [16] confirmed that the knockdown of *MACROD2* inhibited the differentiation of preadipose tissue by suppressing adipogenic genes (*FABP4*, *ADIPOQ*, *CEBPA*, *PPARG2*, and *SREBP1*). Additionally, the genotype distributions of the *MACROD2* rs616996211 and rs6034240 polymorphisms were found to have a significant association with hypertension in the Korean population [17]. However, the exact functional mechanism of *MACROD2* regarding metabolic risk factors is still elusive, and the clinical significance of *MACROD2* rs6110695 has not been well studied.

A significant association between the rs6110695 SNP and total blood cell count was confirmed in this study. The AG genotype group exhibited significantly higher counts of WBC, lymphocyte, monocyte, and granulocyte compared to the AA genotype group. However, the AG genotype group presented a lower % lymphocyte. Interestingly, although the absolute count of all immune cells increased, there was a significant difference in the direction of change in the relative ratio of immune cells. In the Human Protein Atlas (HPA ver. 21.0; http://www.proteinatlas.org (accessed on 15 September 2021)), the *MACROD2* messenger RNA (mRNA) expression level is reported to be enriched in B cells (lymphocytes) and neutrophils (granulocytes) [18]. At this point, altered *MACROD2* function, which its intronic variant, rs6110695 SNP, can induce, may affect the composition of WBCs. However, further research with a larger sample size is needed to identify the precise effect of the rs6110695 A>G polymorphism on immune cells.

The current research showed two differential metabolites between the rs6110695 AA and AG genotype groups. The first one is indoleacrylic acid, which has been reported to be a microbial metabolite of tryptophan [19,20]; unabsorbed tryptophan is catabolized by intestinal microorganisms and converted into indole derivatives, including indoleacrylic acid [21]. Indole derivatives bind to the aryl hydrocarbon receptor (AhR) in intestinal immune cells, inducing increased synthesis of IL-22 and IL-1β release in adipose tissue macrophages via the c-Jun pathway [22,23,24,25]. Furthermore, some tryptophan catabolites in the bloodstream, including indole derivates, have antioxidative and anti-inflammatory effects [21]. Unfortunately, however, other indole derivatives and tryptophan catabolites were not detected; IL-22 was not measured, and no differences in IL-1β were observed between the groups in this study. Therefore, the association between the metabolic pathway involving indoleacrylic acid and the *MACROD2* rs6110695 A>G polymorphism could not be clearly elucidated.

The second differential metabolite is butyrylcarnitine, a member of the acylcarnitines. Mitochondria utilized carnitine (in the form of acyl-carnitine) to uptake long-chain fatty acids (FAs), replenishing the TCA cycle substrate, acetyl-CoA [26]. In other words, acylcarnitines are used as a fuel for cells and completely combust via β-oxidation in mitochondria. In this respect, an increased concentration of circulating fatty acylcarnitines has been considered to be a byproduct of incomplete β-oxidation. Several studies have reported changes in acylcarnitine levels in unhealthy individuals [27,28]. Metabolomics revealed that increased body fat was correlated with incomplete β-oxidation, which primarily resulted in elevated level of short-/medium-chain acylcarnitines [27]. In addition, Zhao et al. [29] demonstrated that increased plasma levels of some acylcarnitine metabolites were associated with a risk of cardiovascular diseases in Chinese individuals with type 2 diabetes. The study participants in the present study did not show clinical indicators of unhealthy status or any disease; thus, we cannot strongly prove the meaning of a butyrylcarnitine level increase at this stage. However, this study showed a significant linear association between the rs6110695 SNP genotype and butyrylcarnitine, and the rs6110695 AG genotype group had significantly higher plasma butyrylcarnitine levels and WBC counts than the rs6110695 AA genotype group. Therefore, we carefully suggest that the increase in butyrylcarnitine levels may precede the onset of unhealthy status, but the direct connection between the *MACROD2* rs6110695 SNP and butyrylcarnitine metabolism remains unclear.

Regarding the metabolite findings, higher plasma indoleacrylic acid and butylcarnitine levels were observed in the AG genotype group than in the AA genotype group in our study; a significant association of these two biomarkers with rs6110695 genotypes was confirmed. As the individuals carrying the rs6110695 AG genotype presented significantly higher WBC counts than those carrying the rs6110695 AA genotype, we suspected that the two major metabolites may be associated with altered immune-related metabolism in the rs6110695 AG genotype group and may be useful as a biomarker for predicting WBC levels in combination with the rs6110695 SNP genotype. Unfortunately, there are a lack of previous studies uncovering a direct link between WBC count and the levels of indoleacrylic acid or butyrylcarnitine to support our findings. Concerning indoleacrylic acid, studies have demonstrated that immune/inflammatory-related diseases are associated with altered levels of indoleacrylic acid [30,31,32,33,34], suggesting a possible indirect connection between WBC count and this metabolite. In the case of butyrylcarnitine, only one study has found a positive association between its levels and WBC count in cerebrospinal fluid in patients with tuberculosis meningitis, which can lead to excessive inflammation [35]. Despite these indirect clues, our ROC curve analysis showed that model 3, consisting of the rs6110695 SNP and the two major metabolites, had a significant predictive ability for WBC count. Although the area under the curve (AUC) value in model 3 did not surpass the commonly accepted threshold of 0.7, indicating adequacy, it still holds meaning for predicting WBC count prediction. This is evident as it demonstrated improved and statistically significant predictive capability compared to models utilizing either the SNP or major metabolites alone. Hence, although the exact mechanisms of these two biomarkers could not be explained, they seem to be potential biomarkers that strengthen WBC count prediction and could thereby be utilized for chronic disease monitoring. However, in vivo and in vitro experiments are required to support these findings and thoroughly explore the exact mechanism.

Notably, the AG genotype group presented a higher WBC-to-apo A-I ratio than the AA genotype group in our study. Likewise, the baseline WBC-to-apo A-I ratio had a substantial relationship with long-term unfavorable outcomes in patients who received percutaneous coronary intervention [36]. Apo A-I is the major lipoprotein component of HDL-cholesterol and primarily regulates its activity [37,38]. The present study speculated that the reduced apo A-I concentration (even if not statistically significant) decreased HDL-cholesterol activity. Indeed, the HDL-cholesterol concentration in the AG genotype group trended toward a decrease compared to that in the AA genotype group (*p* = 0.053) (Table 1), and HDL-cholesterol was significantly correlated with apo A-I (*r* = 0.740, *p* < 0.001) and the WBC-to-apo A-I ratio (*r* = −0.571, *p* < 0.001). Unfortunately, we could not find significant correlations between the major metabolites and apo A-I, HDL-cholesterol, and WBC-to-apo A-I. Thus, the changes in metabolites, including indoleacrylic acid and butyrylcarnitine, seem not to be correlated with apo A-I and its related variables.

Several limitations of the present research should be discussed. First, the sample size of the rs6110695 AG genotype group was too small. Therefore, our results should be interpreted cautiously, and further research with a bigger sample size is required to confirm our results. If the result is replicated in a larger sample size, the two major metabolites identified in this study will be valuable biomarkers for WBC count prediction. Second, since the study sample consisted of Korean participants, it is difficult to generalize the results to individuals of other races. Finally, although different plasma metabolic changes were found, the underlying mechanism of the WBC increase related to the *MACROD2* rs6110695 A>G polymorphism is still elusive; supporting evidence from in vivo and in vitro experimental studies are highly needed.

## 5. Conclusions

Despite the limitations, the multi-omics approach to evaluate WBC count in the Korean population is a rare endeavor in current research, and we finally demonstrated that indoleacrylic acid and butyrylcarnitine significantly differed according to the *MACROD2* rs6110695 genotype. The AUC for the WBC count prediction model, which included the rs6110695 genotype and the two biomarkers, was 0.685 (95% confidence interval: 0.053–0.790), suggesting a potential diagnostic value for WBC, an ideal intermediate phenotype in metabolic disorders. Notably, the model’s prediction ability was significantly enhanced when incorporating genetic variables and major metabolites, compared to models using either the genotype data or major metabolites alone. While it is still premature to apply this model for predicting WBC count, given the AUC is below the typical threshold of 0.7, the present study implies that the rs6110695 SNP genotype and plasma levels of the two metabolic biomarkers can potentially hold promise for managing the future risk of chronic diseases.

## Figures and Tables

**Table 1:** Anthropometric and clinical/biochemical characteristics of the study subjects according to the rs6110695 genotype.

	Total (*n* = 139)				*p*
	AA Genotype (*n* = 129)		AG Genotype (*n* = 10)		
Age (year) ^§^	56.3	±0.59	55.4	±1.48	0.584
Male/Female *n*, (%)	16 (12.4)/113 (87.6)		2 (20.0)/8 (80.0)		0.618
Weight (kg) ^†^	60.6	±0.74	63.6	±1.43	0.051
BMI (kg/m^2^)	24.1	±0.23	24.6	±0.61	0.515
Waist (cm)	86.6	±0.61	87.6	±1.75	0.662
Waist-to-hip ratio	0.90	±0.00	0.90	±0.01	0.827
Systolic BP (mmHg) ^†^	121.6	±1.17	122.8	±3.72	0.763
Diastolic BP (mmHg) ^†^	76.5	±0.83	76.7	±2.33	0.898
Triglyceride (mg/dL) ^†^	141.2	±7.37	159.7	±39.6	0.723
Total cholesterol (mg/dL)	213.5	±2.82	216.8	±13.7	0.760
HDL-cholesterol (mg/dL) ^†^	55.3	±1.38	46.9	±5.26	0.053
LDL-cholesterol (mg/dL)	130.2	±3.08	132.6	±13.9	0.845
Apo A-I (mg/dL) ^†^	153.3	±2.35	140.8	±4.64	0.115
Apo B (mg/dL) ^†^	109.8	±2.41	106.3	±9.55	0.553
WBC-to-apo A-I ratio ^†^	0.03	±0.00	0.05	±0.01	<0.001
Glucose (mg/dL)	92.6	±0.91	89.3	±3.29	0.334
Insulin (μIU/mL) ^§^	8.73	±0.48	6.94	±0.84	0.234
HOMA-IR ^†^	2.01	±0.12	1.49	±0.16	0.214
Adiponectin (ng/mL) ^§^	8.04	±0.42	6.53	±0.90	0.344

Mean ± standard error (SE). ^†^ variables tested following logarithmic transformation. *p* values of the continuous variables were derived from independent *t*-tests, and ^§^ variables were tested with nonparametric tests (Mann–Whitney *U* tests). *p* values of the sex distribution were derived from Fisher’s exact test. All *p* values < 0.05 were considered to indicate significance.

**Table 2:** Total blood cell count and inflammatory markers in the study subjects according to the rs6110695 genotype.

	Total (*n* = 139)				*p*
	AA Genotype (*n* = 129)		AG Genotype (*n* = 10)		
**Total blood cell count**					
WBC (×10^3^/μL) ^†^	4.89	±0.10	6.89	±0.50	<0.001
Lymphocyte count (×10^3^/μL) ^†^	1.73	±0.04	2.10	±0.16	0.018
Monocyte count (×10^3^/μL) ^§^	0.34	±0.02	0.59	±0.10	0.005
Granulocyte count (×10^3^/μL) ^†^	2.82	±0.08	4.21	±0.41	<0.001
Lymphocyte (%)	36.9	±0.67	31.7	±2.16	0.039
Monocyte (%) ^§^	7.99	±0.37	9.16	±1.14	0.229
Granulocyte (%)	55.1	±0.88	59.1	±2.73	0.220
Platelet (×10^3^/μL) ^§^	237.9	±5.99	242.4	±9.98	0.427
MLR ^§^	0.19	±0.01	0.27	±0.03	0.028
GLR ^†^	1.74	±0.06	2.10	±0.27	0.116
PLR ^†^	143.3	±3.58	120.0	±8.43	0.068
MPR ^†^	0.0015	±0.0001	0.0025	±0.0004	0.006
**Inflammatory markers**					
hs-CRP (mg/L) ^§^	1.36	±0.37	0.98	±0.26	0.764
IL-1β (pg/mL) ^§^	0.54	±0.16	0.35	±0.12	0.593
IL-2 (pg/mL) ^§^	47.5	±2.81	63.1	±16.9	0.286
IL-6 (pg/mL) ^§^	3.48	±0.36	3.10	±0.81	0.696
IL-12 (pg/mL) ^§^	16.0	±4.66	6.43	±0.65	0.529
TNF-α (pg/mL) ^§^	7.27	±2.90	4.40	±1.77	0.332
IFN-γ (pg/mL) ^§^	31.6	±26.0	4.08	±0.95	0.973

Mean ± standard error (SE). ^†^ variables tested following logarithmic transformation. *p* values of the continuous variables were derived from independent *t*-tests, and ^§^ variables were tested with nonparametric tests (Mann–Whitney *U* test). All *p* values < 0.05 were considered to indicate significance.

## Data Availability

The data that support the findings of this study are not openly available due to reasons of sensitivity and are available from the corresponding author upon reasonable request.

## Supplementary Materials

The following supporting information can be downloaded at: https://www.mdpi.com/article/10.3390/jpm14080889/s1. Table S1: Anthropometric and clinical/biochemical characteristics in sex-specific subsets according to the rs6110695 genotype. Table S2: Total blood cell count and inflammatory markers in sex-specific subsets according to the rs6110695 genotype. Table S3: UHPLC–MS/MS analysis results according to the rs6110695 genotype. Table S4: UHPLC–MS/MS analysis results in sex-specific subsets according to the rs6110695 genotype. Figure S1: Prediction models for WBC count using rs6110695 genotype and major metabolites in the female subset.
