# Supplementary material for: The MACROD2 rs6110695 A>G Polymorphism and the Metabolites Indoleacrylic Acid and Butyrylcarnitine Potentially Have Clinical Relevance to WBC Count Prediction
Source: J Pers Med. 2024 Aug 22;14(8):889. doi: 10.3390/jpm14080889 (PMC11355238; doi:10.3390/jpm14080889)
Supplement: Supplementary file 1 [file jpm-14-00889-s001.zip › jpm-3139075-supplementary.pdf]

Supplementary information

Supplementary methods

Anthropometric measurements

Body mass index (BMI; kg/m^2) was calculated with height (m^2) and weight (kg) measured using a GL-150 (G-Tech International, Uijeongbu, Korea) and UM0703581 (Tanita, Tokyo, Japan), respectively, with the study participants wearing light clothes and no shoes. The waist-to-hip ratio was calculated with waist circumference (cm) to hip circumference (cm) measured using a plastic measuring tape with measurements to the nearest 0.1 cm; both were measured at the umbilical level and protruding part, respectively. Systolic and diastolic blood pressures (BPs) were measured using a random-zero sphygmomanometer (HM-1101; Hico Medical Co., Ltd., Chiba, Japan) after the subjects rested for at least 10 minutes in a seated position.

Lipid profile measurements

Serum fasting triglyceride, total cholesterol, and high-density lipoprotein (HDL) cholesterol levels were measured using commercial TG, CHOL, and HDL-C Plus kits (Roche, Mannheim, Germany), respectively, following the manufacturer's instructions. Low-density lipoprotein (LDL) cholesterol levels were calculated by the Friedewald formula: LDL-cholesterol = total cholesterol – [HDL-cholesterol + (triglyceride ÷ 5)]. Serum fasting apolipoprotein (apo) A-I and apo B levels were measured via the turbidimetric immunoassay (TIA) method, and the resulting reactions were analyzed using a Cobas 6000 C501 (Roche, Mannheim, Germany).

Glucose and insulin resistance-related marker measurements

Serum fasting glucose levels were measured with the hexokinase method, and the resulting

colorimetric reactions were analyzed via a Hitachi 7600 autoanalyzer (Hitachi, Tokyo, Japan). Serum fasting insulin levels were measured with the electrochemiluminescence immunoassay (ECLIA) method, and the resulting reactions were analyzed via a Cobas 6000 E801 (Roche, Mannheim, Germany). Homeostatic model assessment for insulin resistance (HOMA-IR) was calculated using the following equation: HOMA-IR = insulin × glucose ÷ 405. Plasma adiponectin levels were measured using a Human Adiponectin enzyme-linked immunosorbent assay (ELISA) kit (Otsuka Pharmaceutical Co., Ltd., Tokushima, Japan). The resulting colorimetric reactions were analyzed using a VERSA max microplate reader (Molecular Devices, Sunnyvale, CA, USA).

Total blood cell count measurements

A HORIBA ABX diagnostic analyzer (HORIBA ABX SAS, Parc Euromedicine, Montpellier, France) was used to measure the counts and percentages of white blood cells (WBCs; lymphocytes, monocytes, and granulocytes) and platelet counts. The monocyte-to-lymphocyte ratio (MLR), granulocyte-to-lymphocyte ratio (GLR), platelet-to-lymphocyte ratio (PLR), monocyte-to-platelet ratio (MPR), and WBC-to-apo A-I ratio were calculated using the counts.

Inflammatory marker measurements

High sensitivity-C reactive protein (hs-CRP) levels were measured using a CRPHS reagent kit (Roche, Mannheim, Germany), and the resulting reactions were analyzed using Cobas C502 (Roche, Mannheim, Germany). Interleukin (IL)-1β, IL-6, and tumor necrosis factor (TNF)-α levels were measured using Bio-Plex Reagent kits (Bio-Rad Laboratories, Hercules, CA, USA), and the resulting reactions were analyzed using a Luminex 200 (Luminex Corporation, Austin, Texas, USA). IL-2, IL-12, and interferon (IFN)-γ levels were measured using a Human IL-2 ELISA kit (Cusabio Biotech, Houston, TX, USA), High Sensitivity Human IL-12 (p70) ELISA kit (Genway Biotech Inc, San Diego, CA, USA), and IFN-γ High Sensitivity Human ELISA kit (Abcam plc, Cambridge Science Park, UK), respectively, and the resulting reactions were analyzed using a Victor×5 2030 Multilabel

Plate Reader (PerkinElmer, Hopkinton, MA, USA) to measure the absorbance at 450 nm.

SNP genotyping array, genotyping quality control, and association tests

All equipment and resources required for the Axiom 2.0 assay with automated target preparation are in the Axiom 2.0 Assay Automated Workflow User Guide (P/N 702963). Using the Axiom 2.0 Reagent kit (P/N 901758), approximately 200 ng of genomic DNA was amplified and randomly fragmented into 25 to 125 base pair (bp) fragments. A fragmentation step then further reduced the amplified products to segments of approximately 25-50 bp, which were then end-labeled using biotinylated nucleotides. The samples were then denatured and transferred to the hybridization tray to begin hybridization in the GeneTitan MC Instrument (Affymetrix, Santa Clara, CA, USA). The hybridization step followed the GeneTitan Multichannel Instrument User's Manual (P/N 08-0306) using the Axiom BiobankPlus Genotyping Array KNIHv1.1. After ligation, the arrays were stained and imaged on the GeneTitan MC Instrument (Affymetrix, Santa Clara, CA, USA). The images were then analyzed using Affymetrix GeneChip Command Console Software User Manual (P/N 702569). Genotype data were obtained using the Korean Chip (K-CHIP) available through the K-CHIP consortium. The K-CHIP was designed by the Center for Genome Science, Korea National Institute of Health, Republic of Korea (4845-301, 3000-3031).

PLINK ver. 1.07 (http://zzz.bwh.harvard.edu/plink/) was used for quality control (QC) of the SNP data and to assess associations between WBC counts and SNPs via a linear regression analysis with adjustment for age, sex, and BMI. In QC, samples that had the following characteristics were excluded: gender inconsistency, markers with a high missing rate >0.05, individuals with a high missing rate >0.1, a minor allele frequency (MAF) <0.01, and a significant deviation from Hardy-Weinberg equilibrium (HWE; $p < 1\times10^{-6}$). Additionally, SNPs that were in linkage disequilibrium were excluded. Finally, the remaining 356,375 SNPs and 153 participants were included in our previous work; among them, 139 participants were selected for the present study.

Ultra-high-performance liquid chromatography-tandem mass spectrometry (UHPLC–MS/MS) conditions for metabolic profiling

The column temperature was maintained at 50 °C. A gradient system with two solvents was applied for liquid chromatography (LC). Solvent A was 0.1% formic acid in LC–mass spectrometry (MS) grade water (Thermo Fisher Scientific, Fair Lawn, NJ, USA), and solvent B was 0.1% formic acid in LC–MS grade methanol (Thermo Fisher Scientific, Fair Lawn, NJ, USA). The volumetric change was as shown as follow (Table A).

Table A. The volumetric changes of the mobile phases

RT (min)	A (%)	B (%)
0.0	90.0	10.0
1.0	90.0	10.0
14.0	0.0	100.0
18.0	0.0	100.0
19.0	90.0	10.0
22.0	90.0	10.0

A was 0.1% formic acid solvent in LC-MS grade water, and B was 0.1% formic acid solvent in LC-MS grade methanol.

For MS, ionization was performed with an electrospray ionization (ESI) source, and the data were collected in Full MS-ddMS^2 scan mode with a scan range of 50-1,000 mass-to-charge (m/z). The ESI conditions were set as follows: spray voltage, 3.5 kV; flow rate of nitrogen sheath gas, 40 (arbitrary units); auxiliary gas, 10 (arbitrary units); capillary temperature, 320 °C; S-lens radio frequency level, 50; and auxiliary gas heater temperature, 300 °C. The Full MS conditions were set as resolution, 70,000; maximum IT, 100 ms; and AGC target, 3e^6. For ddMS^2, the following parameters were used: resolution, 17,500; maximum IT, 50 ms; AGC target, 1e^5; isolation window, 2.0 m/z; loop count, 10; and NCE, 30.

Supplementary tables

Table S1. Anthropometric and clinical/biochemical characteristics in sex-specific subsets according to the rs6110695 genotype

	Female (*n*=121)		*p*	Male (*n*=18)*		*p*
	AA genotype (*n*=113)	AG genotype (*n*=8)		AA genotype (*n*=16)	AG genotype (*n*=2)	
Age (year)§	56.1±0.58	55.6±1.85	0.786	57.4±2.42	54.5±1.50	0.327
Weight (kg)	58.9±0.66	63.7±1.81	0.062	72.5±1.93	63.5±0.70	0.118
BMI (kg/m^2)	23.9±0.24	24.9±0.70	0.274	25.4±0.61	23.5±1.26	0.392
Waist (cm)	85.8±0.64	88.6±1.97	0.260	92.1±1.04	83.5±3.00	0.052
Waist-to-hip ratio	0.90±0.01	0.90±0.02	0.902	0.93±0.01	0.91±0.01	0.209
Systolic BP (mmHg)†	120.4±1.23	121.4±4.58	0.812	130.4±3.00	128.0±0.00	0.941
Diastolic BP (mmHg)†	75.7±0.86	74.9±2.48	0.877	82.7±2.19	83.8±3.25	0.837
Triglyceride (mg/dL)§	138.1±8.05	123.4±26.1	0.577	163.5±16.9	305.0±158.0	0.261
Total cholesterol (mg/dL)§	213.6±3.00	203.1±11.3	0.577	212.7±8.42	271.5±32.5	0.078
HDL-cholesterol (mg/dL)	56.5±1.49	51.1±5.56	0.353	46.8±2.89	30.0±5.00	0.118
LDL-cholesterol (mg/dL)	129.8±3.32	127.3±14.5	0.851	133.2±8.41	174.6±0.00	0.353
Apo A-I (mg/dL)†	154.4±2.46	144.3±9.00	0.233	146.1±7.43	127.0±11.0	0.441
Apo B (mg/dL)†	108.6±2.58	101.1±8.38	0.373	117.9±6.51	127.0±40.0	0.721
WBC-to-apo A-I ratio†	0.03±0.00	0.50±0.01	**<0.001**	0.04±0.00	0.06±0.01	0.235
Glucose (mg/dL)	91.6±0.92	90.1±4.07	0.680	99.5±2.95	86.0±3.00	0.078
Insulin (μIU/mL)§	8.70±0.54	6.88±1.06	0.272	8.93±0.67	7.20±0.40	0.368
HOMA-IR†	1.98±0.13	1.49±0.20	0.304	2.19±0.20	1.53±0.03	0.294
Adiponectin (ng/mL)§	8.42±0.45	6.87±1.07	0.364	5.38±0.81	5.16±1.33	0.837

Mean ± standard error (SE). † variables tested following logarithmic transformation. *p values* of the continuous variables were derived from independent *t-tests*, and § variables were tested with nonparametric tests (*Mann–Whitney U* tests). * For the male subset, all variables were tested nonparametrically due to the sample size. *p values* of the sex distribution were derived from *Fisher's exact* test. All *p* values <0.05 were considered to indicate significance.

Table S2. Total blood cell count and inflammatory markers in sex-specific subsets according to the rs6110695 genotype

	Female (n=121)		p	Male (n=18)*		p
	AA genotype (n=113)	AG genotype (n=8)		AA genotype (n=16)	AG genotype (n=2)	
**Total blood cell count**						
WBC (×10³/μL)	4.78±0.10	6.92±0.62	**0.010**	5.61±0.27	6.80±0.85	0.209
Lymphocyte count (×10³/μL)†	1.70±0.04	2.09±0.20	**0.028**	1.93±0.12	2.15±0.10	0.327
Monocyte count (×10³/μL)§	0.32±0.02	0.55±0.12	**0.023**	0.46±0.07	0.73±0.03	0.157
Granulocyte count (×10³/μL)†	2.77±0.08	4.28±0.49	**<0.001**	3.22±0.24	3.93±0.73	0.261
Lymphocyte (%)	37.0±0.70	31.5±2.68	**0.044**	35.8±2.17	32.8±2.75	0.641
Monocyte (%)§	7.87±0.40	8.54±1.33	0.491	8.84±1.07	11.7±0.85	0.392
Granulocyte (%)	55.1±0.93	60.0±3.31	0.176	55.3±2.73	55.6±3.60	0.941
Platelet (×10³/μL)†	242.0±6.60	245.5±9.12	0.630	209.3±10.3	229.8±44.3	0.837
MLR†	0.19±0.01	0.26±0.41	0.083	0.23±0.03	0.34±0.00	0.209
GLR†	1.73±0.62	2.18±0.33	0.107	1.81±2.00	1.81±0.25	0.641
PLR†	147.8±3.84	123.4±9.85	0.088	111.5±5.14	106.1±15.6	0.941
MPR†	0.0014±0.0001	0.0023±0.0005	**0.020**	0.0021±0.0003	0.0033±0.0005	0.157
**Inflammatory markers**						
hs-CRP (mg/L)§	1.38±0.42	1.12±0.31	0.404	1.20±0.29	0.43±0.19	0.294
IL-1β (pg/mL)§	0.40±0.09	0.30±0.14	0.847	1.53±1.08	0.57±0.28	0.837
IL-2 (pg/mL)†	46.2±3.03	47.9±5.53	0.146	56.9±7.19	124.1±86.1	0.732
IL-6 (pg/mL)§	3.28±0.34	3.09±0.98	0.703	4.89±1.58	3.17±1.46	0.941
IL-12 (pg/mL)§	17.0.±5.13	6.84±0.85	0.423	6.65±1.08	5.39±0.00	0.909
TNF-α (pg/mL)§	4.44±0.89	4.81±2.16	0.491	27.2±22.5	2.76±2.75	0.392
IFN-γ (pg/mL)§	35.6±29.8	4.48±1.16	0.821	4.37±1.42	2.48±0.43	0.837

Mean ± standard error (SE). † variables tested following logarithmic transformation. *p values* of the continuous variables were derived from independent *t*-tests, and § variables were tested with nonparametric tests (*Mann–Whitney U* test). * For the male subset, all variables were tested nonparametrically due to the sample size. All *p* values <0.05 were considered to indicate significance.

Table S3. **UHPLC–MS/MS analysis results according to the rs6110695 genotype**

Molecular weight	Molecular formula	HMDB ID	Putative identification	Relative intensity		*p*
				AA genotype (*n*=129)	AG genotype (*n*=10)	
172.085	$C_7H_{12}N_2O_3$	HMDB0011178	Prolylglycine[†]	2715443.4±90731.9	3011940.2±366113.9	0.418
187.062	$C_{11}H_9NO_2$	HMDB0000734	Indoleacrylic acid[§]	77251977.2±6433712.3	127655165.9±19122786.6	**0.037**
188.079	$C_7H_{12}N_2O_4$	HMDB0006029	N-Acetylglutamine[§]	13206898.4±321314.1	14755765.2±1139650.5	0.244
189.079	$C_{11}H_{11}NO_2$	HMDB0029738	Indole-3-methyl acetate[§]	584021.7±199216.8	258167.9±41686.7	0.929
201.136	$C_{10}H_{19}NO_3$	HMDB0000832	Capryloylglycine[§]	727749.7±100998.9	1534609.9±986328.8	0.807
203.116	$C_9H_{17}NO_4$	HMDB0000201	L-Acetylcarnitine[§]	17264908.0±2711346.5	6252414.1±764038.8	0.304
205.131	$C_9H_{19}NO_4$	HMDB0004231	Panthenol[†]	2515890.0±177064.4	1789136.8±251304.0	0.303
214.132	$C_{10}H_{18}N_2O_3$	HMDB0003581	Dethiobiotin[§]	460977.6±29374.3	303927.8±76598.6	0.156
214.157	$C_{12}H_{22}O_3$	HMDB0010727	3-Oxododecanoicacid[§]	225192.0±18307.8	281863.9±75296.1	0.839
216.172	$C_{12}H_{24}O_3$	HMDB0000387	3-Hydroxydodecanoic acid[§]	3201825.7±269364.7	5029469.2±1456525.2	0.293
217.131	$C_{10}H_{19}NO_4$	HMDB0000824	Propionylcarnitine[†]	19475149.9±767412.9	21521080.7±3635611.5	0.549
218.126	$C_9H_{18}N_2O_4$	HMDB0041313	Pantothenamide[§]	419423.0±23383.5	352438.8±76177.8	0.488
230.152	$C_{12}H_{22}O_4$	HMDB0000623	Dodecanedioic acid[§]	569330.1±27217.4	424078.6±54149.1	0.187
231.147	$C_{11}H_{21}NO_4$	HMDB0002013	Butyrylcarnitine[†]	7175510.4±251152.9	9394530.7±1229400.3	**0.025**
255.256	$C_{16}H_{33}NO$	HMDB0012273	Palmitic amide[§]	53525200.7±3561276.2	74659239.9±25934405.9	0.757
259.178	$C_{13}H_{25}NO_4$	HMDB0000756	Hexanoylcarnitine[†]	4188134.4±243630.4	3222005.7±368175.1	0.372
264.111	$C_{13}H_{16}N_2O_4$	HMDB0256432	Phenylacetylglutamine[§]	13445283.6±1021704.3	15277529.2±5482293.2	0.942
281.272	$C_{18}H_{35}NO$	HMDB0002117	Oleamide[§]	54091283.8±5442288.6	97152667.8±32109689.5	0.118
286.220	$C_{20}H_{30}O$	HMDB0000305	Vitamin A[§]	240774.6±14497.9	288198.7±64514.5	0.625
299.282	$C_{18}H_{37}NO_2$	HMDB0002100	Sphingosine[§]	379579.5±35359.8	251845.7±70984.2	0.563
301.298	$C_{18}H_{39}NO_2$	HMDB0000269	Sphinganine[§]	41929639.0±1953907.1	41380523.7±7637219.4	0.751
306.255	$C_{20}H_{34}O_2$	HMDB0002925	Dihomo-γ-linolenic acid[§]	822426.5±50069.1	1107389.6±345904.8	0.463
315.241	$C_{17}H_{33}NO_4$	HMDB0000651	Decanoylcarnitine[§]	18015275.9±1041333.0	17558497.6±2493969.9	0.514

318.219	$C_{20}H_{30}O_3$	HMDB0060053	9-Hydroxyeicosapentaenoic acid[§]	71466.4±5546.9	46705.5±6750.3	0.084
327.314	$C_{20}H_{41}NO_2$	HMDB0013078	Stearoylethanolamide[§]	27047386.0±3331941.8	41460807.0±12796541.4	0.110
355.345	$C_{22}H_{45}NO_2$	HMDB0004080	Arachidoylethanolamide[§]	1002021.5±118082.3	506993.7±212472.7	0.060
362.209	$C_{21}H_{30}O_5$	HMDB0000063	Cortisol[§]	325291.4±13149.5	325691.7±45949.2	0.757
379.249	$C_{18}H_{38}NO_5P$	HMDB0000277	Sphingosine-1-phosphate[†]	3923668.4±132423.4	3998011.3±560717.4	0.953
399.335	$C_{23}H_{45}NO_4$	HMDB0000222	Palmitoylcarnitine[§]	16358019.3±3877878.5	2648720.6±425553.6	0.264
449.314	$C_{26}H_{43}NO_5$	HMDB0000637	Chenodeoxycholic acid glycine conjugate[†]	3420112.3±415598.2	2154865.1±855834.7	0.169
465.309	$C_{26}H_{43}NO_6$	HMDB0000138	Glycocholic acid[§]	1259776.2±175277.9	908362.4±428394.3	0.100
537.513	$C_{34}H_{67}NO_3$	HMDB0004949	Ceramide (d18:1/16:0)[§]	1713204.1±216173.3	2943977.4±887937.3	0.149
584.264	$C_{33}H_{36}N_4O_6$	HMDB0000054	Bilirubin[§]	137259.6±12311.3	272009.5±116649.7	0.987

Mean ± standard error (SE). The relative intensity of each metabolite was normalized by the peak intensity of internal standard (ISTD) prior to statistical analysis. [†] variables tested following logarithmic transformation. *p value*s of the continuous variables were derived from independent *t*-tests, and [§] variables were tested with nonparametric tests (*Mann–Whitney U* tests). All *p value*s are considered to indicate significance.

Table S4. UHPLC–MS/MS analysis results in sex-specific subsets according to the rs6110695 genotype

Putative identification	Relative intensity					
	Female (*n*=121)		***p***	Male (*n*=18)[*]		***p***
	AA genotype (*n*=113)	AA genotype (*n*=113)		AA genotype (*n*=16)	AA genotype (*n*=2)	
Prolylglycine[§]	2659868.6±90114.3	2643225.3±338376.6	0.983	3107940.3±355570.4	4486799.7±326794.3	0.078
Indoleacrylic acid[§]	73851678.9±6970201.2	134111043.9±23339264.2	**0.024**	101266584.2±15554528.9	101831654.0±19232294.1	0.471
N-Acetylglutamine	13314597.9±342825.1	14454331.1±1421680.3	0.397	12446270.9±928099.1	15961501.6±189745.6	0.157
Indole-3-methyl acetate[§]	635664.0±227035.3	249174.7±45380.0	0.884	219298.3±47149.0	294140.5±137553.7	0.549
Capryloylglycine[§]	760999.9±114757.5	595473.9±94347.3	0.573	492920.2±56182.7	5291154.0±5088146.8	0.837
L-Acetylcarnitine[§]	18339861.8±3045580.8	6156252.2±939865.8	0.251	9673046.7±3501216.2	6637061.6±1162030.1	0.837
Panthenol[†]	2523436.0±167804.6	2033732.4±239834.8	0.776	2462596.3±819696.2	810754.0±186537.8	0.078
Dethiobiotin[§]	484593.2±31679.7	352642.5±87922.6	0.251	294192.3±65754.4	109068.7±10827.2	0.209
3-Oxododecanoicacid[§]	221670.8±19824.4	203444.7±64246.9	0.375	250060.8±47839.5	595540.8±128692.9	0.052
3-Hydroxydodecanoic acid[§]	3153758.4±282014.4	4708386.6±1610268.6	0.428	3541300.9±888358.6	6313799.6±4560817.2	0.471
Propionylcarnitine	20047132.2±847686.8	23318470.9±4311562.1	0.335	15435525.0±1176087.7	14331519.7±3110475.5	0.837
Pantothenamide[§]	439998.2±25493.1	399114.2±86726.2	0.739	274110.5±41720.7	165737.2±83459.0	0.471
Dodecanedioic acid[†]	588748.0±29445.8	408389.2±64992.5	0.097	432191.1±61740.8	486836.3±93540.5	0.732
Butyrylcarnitine[†]	7399536.9±275894.8	10051935.3±1447738.3	**0.025**	5593323.1±367803.0	6764912.3±827051.3	0.261
Palmitic amide[§]	55163714.5±3990078.3	48924141.4±9850699.7	0.566	41953196.9±4777507.2	177599633.7±119598418.1	0.118
Hexanoylcarnitine[†]	4299940.5±271083.9	3123586.7±428262.2	0.285	3398503.7±401294.1	3615681.6±877904.4	0.732
Phenylacetylglutamine[§]	14350216.2±1118189.7	18569866.9±6367958.9	0.588	7054197.4±1665911.0	2108178.7±533190.1	0.392
Oleamide[§]	55975376.0±6123385.3	87737134.2±38039699.9	0.364	40784882.8±6855417.0	134814802.2±64084914.6	0.078
Vitamin A[§]	223427.0±14324.7	222151.2±59731.9	0.794	363291.5±49978.9	552388.5±12202.4	0.327
Sphingosine[§]	401494.1±38547.5	278076.8±87119.1	0.566	224807.1±76429.8	146921.2±21672.6	0.837
Sphinganine[§]	43134788.9±2151049.2	43491995.7±9484036.4	0.754	33418268.5±3626320.1	32934635.9±4127910.9	0.549
Dihomo-γ-linolenic acid[§]	753723.5±39628.8	1101318.3±434668.8	0.851	1307641.4±267848.9	1131674.6±305421.3	0.732

Decanoylcarnitine†	18303378.9±1165479.1	16669647.5±3050497.7	0.608	15980548.2±1630949.1	21113897.6±1881161.1	0.261
9-Hydroxyeicosapentaenoic acid§	69945.4±5943.3	40152.9±2089.2	**0.028**	82208.4±15663.2	72915.7±32704.1	0.732
Stearoylethanolamide§	26831807.7±3700426.7	44981481.9±15921913.4	0.133	28569907.4±6445468.9	27378107.4±4765294.0	0.837
Arachidoylethanolamide§	1051347.0±127591.1	558264.4±265768.8	0.065	653660.2±302654.9	301910.8±34788.3	0.837
Cortisol§	323543.3±14381.6	304941.7±55508.6	0.810	337637.8±31270.0	408691.7±11178.9	0.549
Sphingosine-1-phosphate†	3999645.1±146509.2	4107134.5±704518.2	0.998	3387083.4±229439.4	3561518.6±111080.7	0.641
Palmitoylcarnitine§	17328453.0±4306738.3	2261997.6±212093.3	0.101	9504331.0±7275840.5	4195612.7±1974507.3	0.549
Chenodeoxycholic acid glycine conjugate†	3112783.7±418327.3	1431859.0±517052.1	0.098	5590620.6±1515422.4	5046889.1±3875569.0	0.837
Glycocholic acid†	1146891.7±154237.6	829801.8±501971.1	0.134	2057023.0±900465.2	1222604.7±1039106.5	0.641
Ceramide (d18:1/16:0)§	1687326.0±233856.2	2285441.8±954896.1	0.453	1895968.0±573257.1	5578119.9±1129135.2	0.078
Bilirubin§	135966.3±13515.2	274039.1±146023.1	0.353	146393.7±28092.0	263891.1±123299.4	0.209

Mean ± standard error (SE). The relative intensity of each metabolite was normalized by the peak intensity of internal standard (ISTD) prior to statistical analysis. † variables tested following logarithmic transformation. *p values* of the continuous variables were derived from independent *t*-tests, and § variables were tested with nonparametric tests (*Mann–Whitney U* tests). * For the male subset, all variables were tested nonparametrically due to the sample size. All *p values* are considered to indicate significance. Information on putatively identified metabolites, including molecular weight, molecular formula, and HMDB ID can be found in Table S3.

Supplementary figure

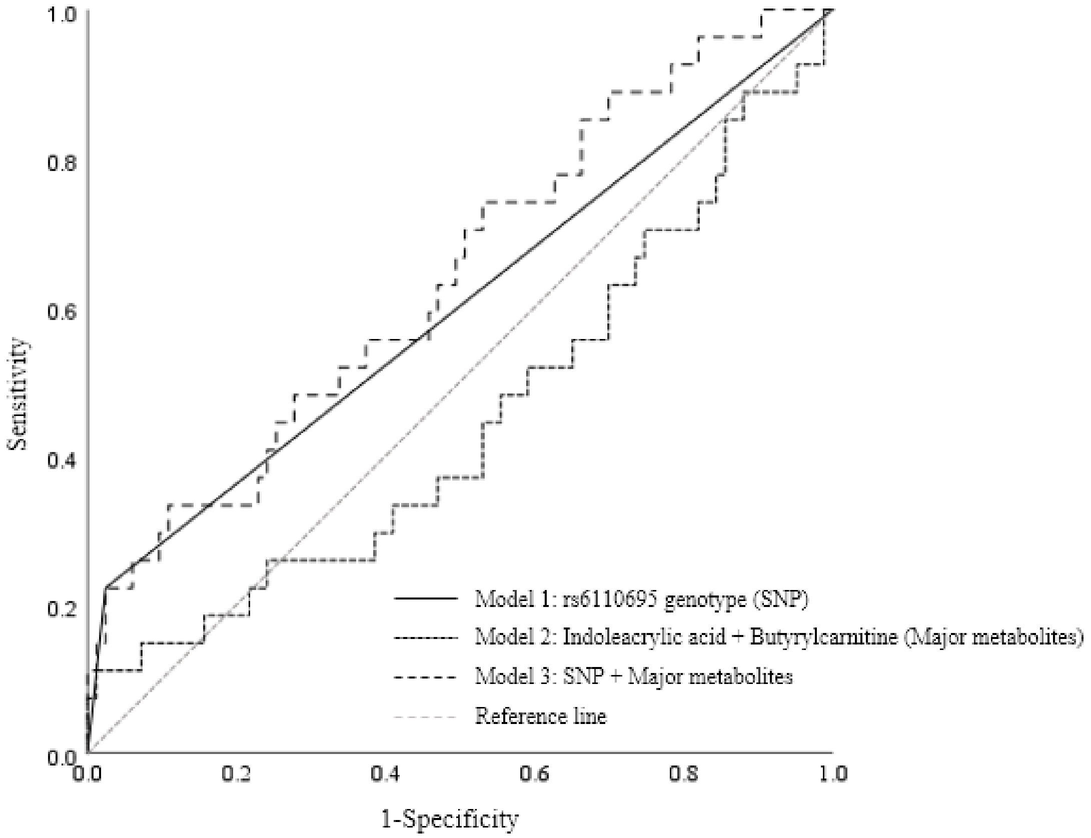

Model	AUC	SE	95% CI		Significance
			Lower	Upper	
Model 1	0.599	0.068	0.466	0.732	0.123
Model 2	0.449	0.067	0.317	0.581	0.426
Model 3	0.643	0.062	0.522	0.764	**0.026**

Figure S1. Prediction models for WBC count using rs6110695 genotype and major metabolites in the female subset.

AUC: area under the curve. CI: confidence interval. SE: standard error.
